# A Case of Intracardiac Thrombus in a Recovery Coronavirus Disease 2019 (COVID-19) Patient

**DOI:** 10.7759/cureus.20972

**Published:** 2022-01-05

**Authors:** William Lim, Swann Tin, May Breitling, Richard Grodman, Keith Diaz

**Affiliations:** 1 Internal Medicine, Richmond University Medical Center, New York, USA; 2 Cardiology, Richmond University Medical Center, New York, USA; 3 Pulmonary and Critical Care, Richmond University Medical Center, New York, USA

**Keywords:** sar-cov 2 infection, covid-related hypercoagulability, thrombo embolic disease, intracardiac thrombus, covid-19

## Abstract

Coronavirus disease 2019 (COVID-19) was thought to mainly affect the respiratory system. However, studies have shown that it can be associated with hypercoagulability leading to thromboembolism. Although venous thromboembolism is a common complication associated with COVID-19, arterial thrombosis and intracardiac thrombosis are not frequently described. We herein report a case of a 54-year-old male with a past medical history of end-stage renal disease, diabetes mellitus, hypertension, heart failure, chronic obstructive pulmonary disease who presented to the emergency department with shortness of breath and was found to have intracardiac thrombus in post-recovery COVID-19 state.

## Introduction

In December 2019, a new respiratory virus called severe acute respiratory virus syndrome (SARS-CoV-2) was first discovered in China. The disease caused by SARS-CoV-2 was named coronavirus disease 2019 (COVID-19) [1] and it rapidly spread across the countries, resulting in a global pandemic. COVID-19 was thought to mainly affect the respiratory system. However, studies have shown that it can be associated with hypercoagulability leading to thromboembolism. Although venous thromboembolism is a common complication associated with COVID-19, arterial thrombosis and intracardiac thrombosis are not frequently described [2-4]. Herein, we report a case of a patient presenting to the emergency department with shortness of breath after recovering from COVID-19 a month ago and was found to have intracardiac thrombus.

## Case presentation

A 54-year old male with a past medical history of end-stage renal disease with hemodialysis on Tuesday, Thursday, and Saturday, diabetes mellitus, hypertension, heart failure, chronic obstructive pulmonary disease presented to the emergency department with shortness of breath for two days. The patient was admitted to the hospital for coronavirus disease 2019 (COVID-19) pneumonia one month ago and was discharged with 4 liters of nasal cannula oxygen. On this current visit, the patient’s vital signs were as follows; temperature: 98° F, blood pressure: 150/89 mmHg, pulse rate: 97/min, respiratory rate: 28/min, and oxygen saturation 95% with a non-rebreather mask. His coagulation profile showed PT of 13.7 s, INR of 1.1, and PTT of 30.2 s. Laboratory studies were notable for the following: hemoglobin 6.5 g/dl, blood urea nitrogen 70 mg/dl, creatinine 9.6 mg/dl, and pro-BNP 40000 pg/ml. The patient stated that he had intermittent dark stools after discharge from the hospital. Pantoprazole 40 mg intravenous (IV) twice a day was started and a total of 4 units of packed red blood cells (PRBCs) were transfused throughout the hospital stay, after which hemoglobin went up to 9.5 g/dl.

**Figure 1:**
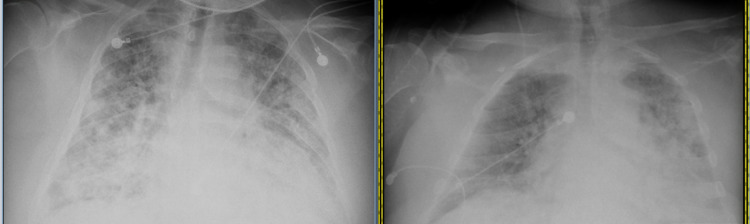
Chest X-ray showing worsening of infiltrates (A) from prior visit study (B)

**Figure 2:**
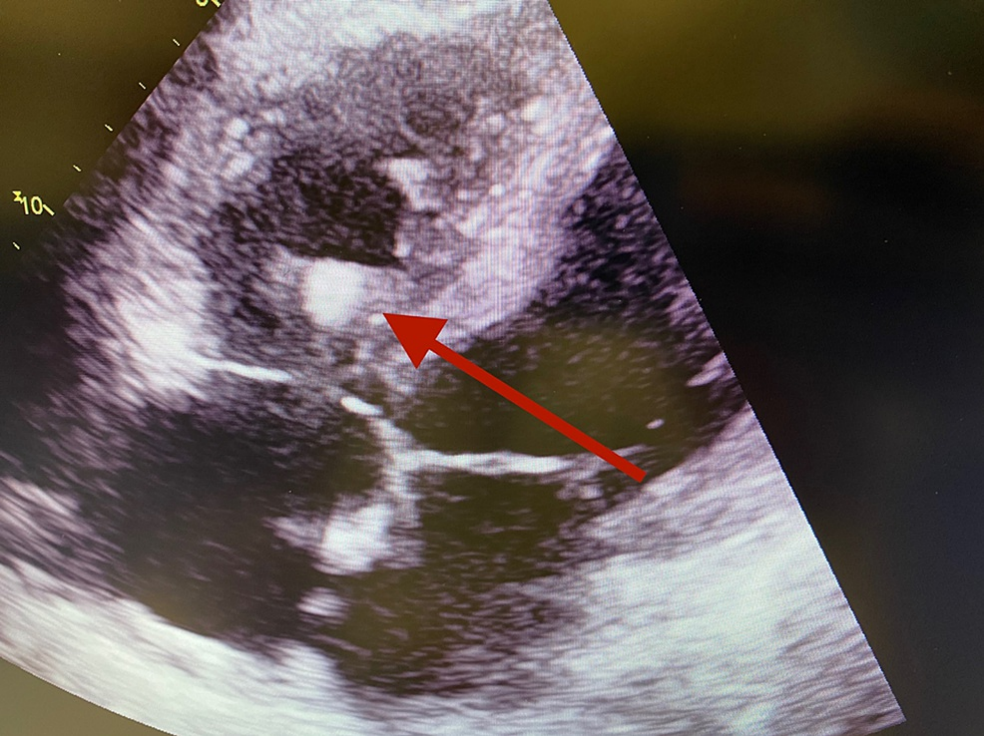
Echocardiogram showing intracardiac thrombus in right ventricle on subcostal view

Chest X-ray showed worsening of infiltrates from the prior visit study (Figure 1). An echocardiogram revealed mild to moderate global left ventricular hypokinesis with an ejection fraction of 45-50%, grade 2 diastolic dysfunction, and a large (1.1 X 1.5 cm) dense oval very highly mobile thrombus seen in the right ventricular cavity near the base (Figure 2). CT angiogram of the chest obtained to rule out pulmonary embolism came out negative. After endoscopy and colonoscopy revealed no source of bleeding, a heparin drip was started and the patient was transitioned to apixaban upon discharge for intracardiac thrombus. After a few rounds of hemodialysis throughout the hospital stay, the patient's respiratory status improved and the patient was discharged from the hospital.

## Discussion

The clinical features of COVID-19 infection range from asymptomatic infection to critical and fatal illness. Cough, myalgias, and headaches are the most commonly reported symptoms. Other less commonly described symptoms include diarrhea, sore throat, and loss of smell or taste. COVID-19 mainly affects the respiratory system resulting in fever, cough, dyspnea, and pneumonia. Acute respiratory distress syndrome (ARDS) is the most common complication in severe disease [5,6]. Other commonly described complications include thromboembolic events, acute cardiac injury, kidney injury, and inflammatory complications. 

Although COVID-19 is known to be associated with hypercoagulability, the pathophysiology behind it is still unclear. The proposed mechanisms include complement-mediated endothelial injury, venous stasis, and coagulation abnormalities. The contribution of complement-mediated endothelial injury has been suggested due to the activation of the alternative complement pathway by SARS-CoV-2 spike protein [7,8]. Furthermore, COVID-19-induced inflammatory response, including cytokines such as interleukin-6 (IL-6) and other cytokines, promote coagulability by mediating endothelial injury. Dysfunction of endothelial cells induced by COVID-19 infection leads to excess thrombin generation and fibrinolysis shutdown [9]. Immobilization can lead to stasis of blood flow in all critically ill patients, regardless of whether they have COVID-19. The hypothesis of coagulation abnormalities' contribution to hypercoagulability is based on increased levels of circulating prothrombotic factors found in COVID-19 such as elevated factor VIII, fibrinogen, circulating prothrombotic microparticles, and neutrophil extracellular traps (NETs) [10-12]. The hypoxia observed in severe COVID-19 can also stimulate thrombosis by increasing blood viscosity or by activation of the hypoxia-inducible factor-1 (HIF-1) signaling pathway leading to increased blood viscosity [13]. Studies have shown that COVID-19 related coagulopathy and complications lead to longer hospitalizations and death, and on the other hand, critically-ill patients often show an increased thrombotic diathesis (with higher D-dimer and lower fibrinogen values) [14-16]

An observational study reported that anticoagulation may be associated with improved outcomes in patients hospitalized with COVID-19 [17]. However, current guidelines do not recommend post-discharge prophylactic anticoagulation for COVID-19 unless patients have documented thromboembolism or have major prothrombotic risk factors [18]. Nevertheless, physicians should have a high index of suspicion of thromboembolic complications in recovering COVID-19 patients. 

## Conclusions

This case is distinctive because the discovery of intracardiac thrombus was in a patient recovering from COVID-19 illness as opposed to patients being acutely ill with COVID-19 as in other cases. Hypercoagulability associated with COVID-19 is a growing field of research. This case highlights and adds to a growing body of literature regarding anticoagulant use and thromboembolic complications in recovering COVID-19 patients. 
